# Spill-in counts in the quantification of ^18^F-florbetapir on Aβ-negative subjects: the effect of including white matter in the reference region

**DOI:** 10.1186/s40658-019-0258-7

**Published:** 2019-12-19

**Authors:** Francisco Javier López-González, Alexis Moscoso, Nikos Efthimiou, Anxo Fernández-Ferreiro, Manuel Piñeiro-Fiel, Stephen J. Archibald, Pablo Aguiar, Jesús Silva-Rodríguez, for the Alzheimer’s Disease Neuroimaging Initiative

**Affiliations:** 1 Molecular Imaging and Medical Physics Group, Radiology Department, Faculty of Medicine, Universidade de Santiago de Compostela, Galicia, Spain; 2 Nuclear Medicine Department and Molecular Imaging Research Group, University Hospital (SERGAS) and Health Research Institute of Santiago de Compostela (IDIS), Santiago de Compostela, Galicia Spain; 3 PET Research Centre, Faculty of Health Sciences, University of Hull, Hull, UK; 4 Pharmacy Department and Pharmacology Group, University Hospital (SERGAS) and Health Research Institute Santiago Compostela (IDIS), Santiago de Compostela, Galicia Spain; 5 R&D Department, Qubiotech Health Intelligence SL, A Coruña, Galicia Spain

**Keywords:** Amyloid, PET, Quantification, SUV, PVE

## Abstract

**Background:**

We aim to provide a systematic study of the impact of white matter (WM) spill-in on the calculation of standardized uptake value ratios (SUVRs) on Aβ-negative subjects, and we study the effect of including WM in the reference region as a compensation. In addition, different partial volume correction (PVC) methods are applied and evaluated.

**Methods:**

We evaluated magnetic resonance imaging and ^18^F-AV-45 positron emission tomography data from 122 cognitively normal (CN) patients recruited at the Alzheimer’s Disease Neuroimaging Initiative (ADNI). Cortex SUVRs were obtained by using the cerebellar grey matter (CGM) (SUVR_CGM_) and the whole cerebellum (SUVR_WC_) as reference regions. The correlations between the different SUVRs and the WM uptake (WM-SUVR_CGM_) were studied in patients, and in a well-controlled framework based on Monte Carlo (MC) simulation. Activity maps for the MC simulation were derived from ADNI patients by using a voxel-wise iterative process (BrainViset). Ten WM uptakes covering the spectrum of WM values obtained from patient data were simulated for different patients. Three different PVC methods were tested (a) the regional voxel-based (RBV), (b) the iterative Yang (iY), and (c) a simplified analytical correction derived from our MC simulation.

**Results:**

WM-SUVR_CGM_ followed a normal distribution with an average of 1.79 and a standard deviation of 0.243 (13.6%). SUVR_CGM_ was linearly correlated to WM-SUVR_CGM_ (*r* = 0.82, linear fit slope = 0.28). SUVR_WC_ was linearly correlated to WM-SUVR_CGM_ (*r* = 0.64, linear fit slope = 0.13). Our MC results showed that these correlations are compatible with those produced by isolated spill-in effect (slopes of 0.23 and 0.11). The impact of the spill-in was mitigated by using PVC for SUVR_CGM_ (slopes of 0.06 and 0.07 for iY and RBV), while SUVR_WC_ showed a negative correlation with SUVR_CGM_ after PVC. The proposed analytical correction also reduced the observed correlations when applied to patient data (*r* = 0.27 for SUVR_CGM_, *r* = 0.18 for SUVR_WC_).

**Conclusions:**

There is a high correlation between WM uptake and the measured SUVR due to spill-in effect, and that this effect is reduced when including WM in the reference region. We also evaluated the performance of PVC, and we proposed an analytical correction that can be applied to preprocessed data.

## Background

Brain amyloidosis is one of the best-defined biomarkers for Alzheimer’s disease (AD), playing a central role in the novel National Institute on Aging and Alzheimer’s Association (NIA-AA) AT(N) framework [1]. Positron emission tomography (PET) using fibrillary amyloid-β (Aβ PET) tracers is one of the main methodologies to assess this biomarker, together with cerebrospinal fluid (CSF) Aβ42 and Aβ42/Aβ40 measurements. In its simplest form, Aβ PET can be classified as positive/negative through visual inspection [2] or by applying a threshold to an image-derived parameter [3]. Despite Aβ deposition in the grey matter (GM) occurs on a continuum, categorical classification of individual subjects is relevant for clinical diagnosis [4], for the inclusion of subjects in therapeutic trials [5], and for distinguishing Aβ-dependent and independent changes in brain cognition, structure, and function [6].

The most used image-derived parameter in Aβ PET is the SUVR (standardized uptake value ratio) between a target region, a compound of cortex regions known to be involved in AD, and a reference region. The choice of an optimal reference region implies taking into account some theoretical requirements, such as to have similar perfusion characteristics to the target region, to be free of specific binding sites (for the particular case, free of Aβ plaques) and to have a non-specific binding similar to the target region [7], among others. The cerebellar GM (CGM) has been traditionally used as a reference region for the quantification of Aβ PET, since it fulfills most of these criteria, with the exception of Aβ plaques appearing in the CGM at advanced stages of the disease [8] and in some genetic variants of AD [9]. Regarding this, recent studies highlighted that the effect of cerebellar Aβ pathology on SUVR quantification would be negligible, even in subjects with high cortex Aβ burdens [10]. Nevertheless, recent publications have pointed that alternative regions such as the whole cerebellum (WC), including both GM and white matter (WM) [11, 12], or the whole brain WM alone as a reference region [13–15], could provide improved results when compared with CGM. Including WM into the reference region could have some potential advantages, such as (a) the WM is a high-uptake region, which will lead to higher voxel count-rates and thus, less variability in the measurement of the reference region mean values used to calculate SUVR [16]; (b) WM is a large region, providing a better resistance to small registration errors [17]; and (c) WM is usually centered on the field-of-view, being less affected by scatter correction errors that usually occur near the edge of the scanner [14]. On the other hand, WM does not fulfill the aforementioned criteria for a suitable reference region. In particular, WM has significant non-specific binding compared to the cortex GM [18], and the underlying mechanism of Aβ uptake in the WM for the different radiotracers is poorly understood [19]. Some authors have suggested that it could to be related to increased tracer lipophilicity or to specific binding to β-sheet structured myelin basic proteins [20] and as such, related to age-associated demyelination. Furthermore, recent studies have suggested that WM uptake might be more relevant to the evolution of the disease than previously expected [19], with WM uptake increasing with age and with disease progression.

Thus, the good performance of these alternative reference regions including WM remains an interesting field of exploration. An important fact to take into account when discussing Aβ PET quantification is that it can be further hardened by the limitations of PET imaging [7]. One of the most relevant problems regarding Aβ PET images is the partial volume effect (PVE), defined as the spill-over of counts between different image regions due to the limited spatial resolution of the PET images [21]. The PVE is often regarded as two separate effects: the counts that go from the target region to adjacent regions (spill-out) and the counts that go from these adjacent regions into the target region (spill-in). These PVEs are proportional to the differences in uptake between the target and the adjacent region (contrast), being more prominent from hot to cold regions [22], and to the size of the regions, as, in relative terms, small regions are more sensitive to PVE than larger regions [23]. Thus, two different effects must be considered in the estimation of SUVR from Aβ PET images. First, the spill-out activity from the GM to the WM and CSF due to cortical thickness and its variations due to atrophy in longitudinal studies. This effect has been broadly studied, concluding that different PVE corrections (PVC) provide more consistent longitudinal results [24, 25]. Second, the spill-in activity from the WM into the GM, which might be especially relevant for studies where primarily amyloid-negative patients are used, such as in the calculation of SUVR thresholds. In a recent study [26], a visual inspection of SUVR borderline false-positive cases was related to high radiotracer retention in WM. In addition, other publications [27] reported a correlation between white and gray matter uptake in ^11^C-Pittsburgh compound B (^11^C-PIB) in cognitively normal (CN) patients, and how this correlation disappeared after applying PVC. Spill-in in Aβ-negative patients have also been evaluated focusing on its effect on kinetic parameters, demonstrating a significant bias of the non-specific binding components in GM due to WM spill-in both in ^18^F-based and ^11^C-based tracers [28, 29]. These latter findings could explain why reference regions containing WM seem to be more robust, as the inclusion of WM counts into the reference region could partially compensate for this effect.

In this work, we aim to provide a systematic study of the impact of WM spill-in activity on the calculation of SUVR values when using different reference regions with and without WM. For this, we present a MC methodology for simulating realistic amyloid PET studies. We also propose a simple analytical correction.

## Methods

### Patient cohort

Patient data used in the preparation of this article were obtained from the Alzheimer’s disease Neuroimaging Initiative (ADNI) database [30]. ADNI was launched in 2003 as a public-private partnership, led by Principal Investigator Michael W. Weiner, MD. The primary goal of ADNI has been to test whether serial magnetic resonance imaging (MRI), PET, other biological markers, and clinical and neuropsychological assessment can be combined to measure the progression of mild cognitive impairment (MCI) and early AD. In this work, we included 122 CN patients recruited at the start of ADNI2 who underwent a baseline structural MRI and ^18^F-AV-45 PET scan.

### Image acquisition and preprocessing

PET images were acquired by using dynamic 3D acquisitions of 5-min frames from 30 to 60 min after the injection of 370 MBq of ^18^F-AV-45. Every image was reviewed for protocol compliance by the ADNI PET Quality Control team. Currently, there are four types of processed PET image data available for download the ADNI database [31]:

- Co-registered dynamic: the acquired dynamic frames are recombined into a co-registered dynamic image set co-registering frames two to four to the first frame to avoid movement artifacts.
- Co-registered averaged: 30 min static image obtained averaging the frames on the previously described dynamic image.
- Co-registered averaged images standardized: each subject’s co-registered averaged image is reoriented into a standard template
- Co-registered averaged images standardized and smoothed: the above-mentioned images are filtered with a scanner-specific filter function (can be a non-isotropic filter) to produce images of a uniform isotropic resolution of 8 mm FWHM.

For carrying out this work, images in preprocessing level (b) were downloaded. Any extra processing was performed in-house as detailed in the following sections.

### ^18^F-AV-45 PET quantification

**Fig. 1:**
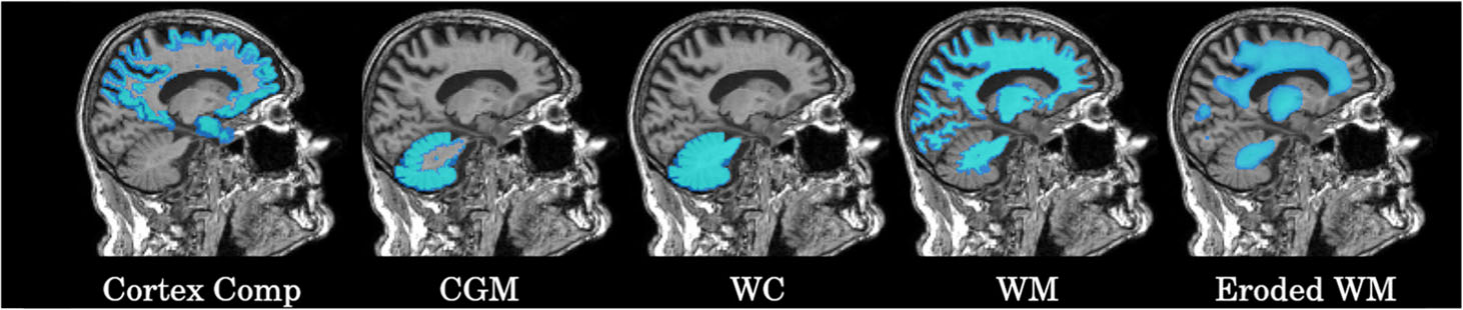
Different ROIs used for the quantification of ^18^F-AV-45 PET. From left to right: cortex composite region, CGM, WC, WM, and eroded WM ROIs

Image processing was performed using the Statistical Parametric Mapping (SPM) software package version 12 [32]. PET and MRI images were co-registered using the MRI image as the reference space. MRI images were segmented into GM, WM, CSF, bone and soft tissue, and normalized to the Montreal Neuroimaging Space (MNI), by using the Local Adaptative Segmentation (LAS) and the Diffeomorphic Anatomical Registration Through Exponentiated Lie Algebra (DARTEL) normalization [33] tools provided by the Computational Anatomy Toolbox [34, 35]. The inverse of the normalization transformation matrix was used to take the Hammersmith atlas [36] back into the native MRI space. Voxel tissue probability maps generated by the segmentation were binarized to generate GM, WM, and CSF masks by using a GM favoring approach. Voxels in the GM-WM and GM-CSF interfaces were considered GM when GM probability was > 0.1 unless WM (or CSF) probability was bigger than 0.5 [37]. The same approach was applied in the CSF-WM interface, favoring CSF over WM. The inverted Hammersmith atlas was multiplied by the GM mask to generate a GM patient-specific atlas. WM and CSF were added to the patient-specific atlas as uniform tissues (results of the different steps of the image processing are shown in Additional file 1: Supplementary Figure S1). PET images were smoothed to achieve a uniform isotropic resolution of 8 mm FWHM with the scanner-dependent smoothing values provided by ADNI (see Additional file 1: Supplementary Table S1) when required. The average cortex uptake was measured by using a composite region of interest (ROI) integrating the GM from the anterior and posterior cingulate, the precuneus, and the frontal, lateral temporal, and lateral parietal cortex. The WM mask was used as a ROI to calculate the average WM uptake. We also used an eroded WM ROI, generated as described in previous works [16]. Cortex SUVR values were obtained by using both the CGM (SUVR_CGM_) and the WC (GM + WM) (SUVR_WC_) as reference regions. An example of the different ROIs used in the quantification process is shown in Fig. 1. WM and eroded WM uptakes were normalized by the CGM value (WM-SUVR_CGM_). The measured SUVR values were compared, when possible, with those provided by the ADNI PET Core at Berkeley [38], for validation purposes (see Additional file 1: Supplementary Figure S2).

### Relation between WM-SUVR and cortex SUVR

Firstly, the variability of WM-SUVR_CGM_ across CN amyloid-negative subjects was studied after removing those CN patients categorized as amyloid-positive by the 1.11 SUVR_WC_ ADNI threshold. The histogram of WM-SUVR_CGM_ values was then fitted to a normal distribution. The correlation between cerebellum WM (inside the reference region) and the rest of the WM were evaluated to ensure that the inclusion of cerebellar WM into the reference region could positively compensate for WM spill-in, as suggested by our hypothesis. Then, the relation between cortex SUVR (SUVR_CGM_ and SUVR_WC_) and WM-SUVR_CGM_ measurements was assessed. This analysis was performed by using both the whole WM and the eroded WM as defined by ADNI.

### Monte Carlo simulation

The impact of the changes in WM uptake on the quantification of cortex SUVR was evaluated in a well-controlled framework using Monte Carlo (MC) simulations. To this end, simulated ^18^F-AV-45 PET images were generated using realistic activity maps and widely validated MC simulation techniques:

#### Generation of realistic activity maps

**Fig. 2:**
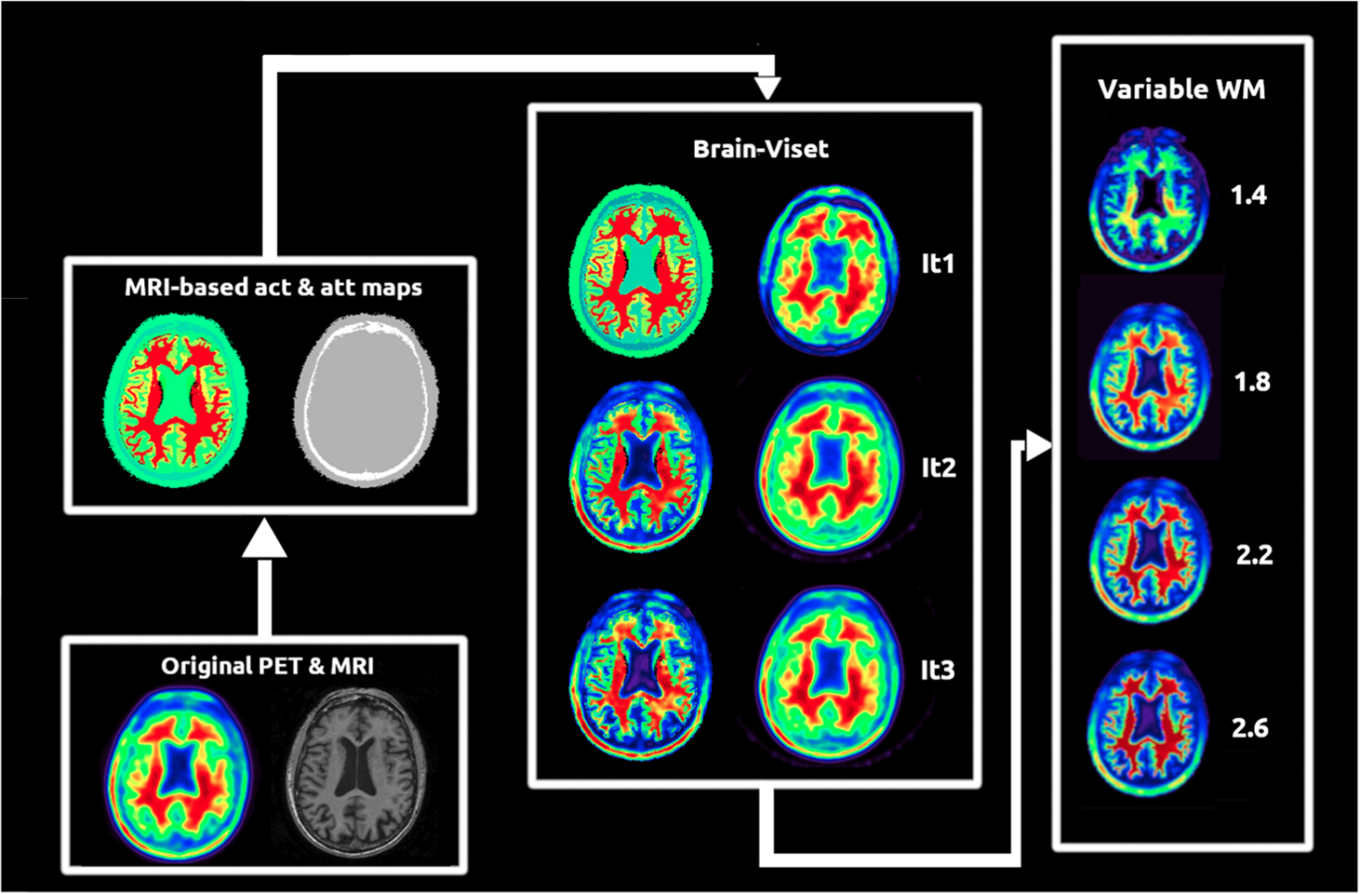
Layout of the BrainViset procedure used for the generation of realistic activity maps for our MC simulation

Patient-specific activity maps were generated by using the BrainViset (voxel-based iterative simulation for emission tomography) iterative procedure, which is explained in detail elsewhere [39]. In brief, initial activity and attenuation maps were generated by filling the patient-specific atlas with activity values from the original PET image and with the corresponding attenuation values for each of the segmented tissues (see Additional file 1: Supplementary Figure S1). After MC simulation, the reconstructed images were compared voxel-wise with the corresponding ADNI PET studies in an iterative process where the activity inputs maps were being modified at each iteration until the correlation coefficients between the original ADNI images and the simulated images were ≥ 0.99. This procedure was performed for five amyloid-negative patients acquired using a GE Discovery STE scanner (ADNI IDs 4579, 4580, 4254, 4276, 4421). After obtaining the activity maps for each patient, 10 different ground truth WM-SUVR_CGM_ values were introduced into the obtained activity maps in order to cover all the spectrum of WM-SUVR_CGM_ derived from the patient data. The theoretical SUVR_CGM_ was maintained (SUVR_WC_ values were not constant as our WM variability included WM on the cerebellum). This resulted in 50 activity maps (10 per patient) and 5 attenuation maps (1 per patient) as inputs for our MC simulation. A schematic view of the activity map generation process is shown in Fig. 2.

#### Simulated data

MC simulations were performed using the open-access package SimSET (v.2.9.2) (simulation system for emission tomography) [40–42]. SimSET includes detailed physics simulation (positron range and non-colinearity, photoelectric effect, coherent scattering, and incoherent scattering) for energies of interest in nuclear medicine (below 1 MeV). The scanner MC model for the GE Discovery STE scanner was implemented as presented by previous works [43]. The model was tested using the NEMA NU-2007 protocol. Simulated NEMA results were validated over the clinical scanner present at CIMES (Centro de Investigaciones Medico Sanitarias, Fundación Universidad de Málaga). In all the NEMA sections, the percentage error was found below 10%.

The simulation times were adjusted to replicate the 20-min total acquisition times in the ADNI protocols. Since the PET scanner was modeled as a solid cylinder of BGO, the simulation times were adjusted in order to match the sensitivities by using the NEMA sensitivity test results. The simulations were performed on a desktop computer including an Intel® Core™ i7-4790K CPU providing 8 cores at 4.00 GHz each (Intel Corporation, Santa Clara, CA, USA) and 32 GB of DDR4 RAM. Each simulation was divided into eight sub-processes in order to use the eight threads of the processor. Each simulation consumed around 8 h of CPU time.

#### Image reconstruction

The image reconstruction of the simulated data was performed with the ordered subset expectation maximization (OS-EM) [44] as implemented in STIR (Software for Tomographic Image Reconstruction) [45, 46]. STIR is an Open Source PET reconstruction toolkit maintained by the University College London (UCL). For more information, head to the STIR wiki [47]. Reconstruction parameters were set to fit those of the scanner. Five full iterations were performed (35 sub-iterations, 7 subsets). Neither post-filtering nor inter-iteration filtering was applied. The matrix and voxel size of the reconstructed images were 128 × 128 × 47 and 1.95 mm × 1.95 mm × 3.27 mm.

### Simulated data analysis

SUVR_CGM_, SUVR_WC_, and WM-SUVR_CGM_ for the simulated data were calculated as described below for the patient data. Data was analyzed for PVC and non-PVC data. For each case, a single fit with a unified slope and variable intercept for each of the simulated SUVRs was obtained by using a general linear model. The independence of the slope for each individual subject with the simulated SUVR was tested by using a linear mixed model and introducing a random term to assess this dependency.

### Partial volume correction

Two PVC methods were tested on the simulated data: (a) the region-based voxel-wise (RBV) and (b) the iterative Yang (iY) methods. The corrections were applied without applying the smoothing to 8-mm included in the ADNI processing, directly convolving by the point-spread function (PSF) calculated for our MC simulation model. The corrections were performed by using the PETPVC toolbox [48]. This open-source package provides the necessary tools for applying a wide range of PVC methods. The toolbox is developed using C++ and optimized for fast execution times.

For the applied corrections, a segmented PET image is used as an input for the PSF deconvolution (see Additional file 1: Supplementary Figure S1). The segmented PET consists of an MRI-derived personalized atlas (generated as defined in the SUVR quantification section) filled with ROI values based on the PET image.

The RBV PVC [25] is an extension of the popular geometrical transfer matrix (GTM) method and the voxel-wise correction of Yang et al. [49]. The mean ROI activity values are calculated using the GTM, and then a voxel-by-voxel correction is performed in order to produce a corrected image by performing a voxel-wise multiplication of the uncorrected PET image with a PVE correction factor. This factor relates PVE corrected ROI values obtained by GTM with smoothed segmented PET voxel values. RBV was chosen for this work before the original GTM since it provides a corrected image, which facilitates the calculation of corrected SUVR values using the same methodology used for uncorrected images. In addition, RBV accounts for within-compartment variability, preventing biases in GM PVC corrected values due to WM variability.

The iY method [50] extends the aforementioned voxel-wise Yang method [49] process. In contrast with RBV, instead of calculating the regional mean values via the GTM, the values are estimated from the PET data itself. The Yang correction is applied and the mean value estimates are recalculated. This process is iterated several times (for this work, we used 10 iterations), updating the PET image at each iteration with the input of the previous iteration, providing more accurate correction factors.

### Linear correction of WM spill-in

In addition, a simple analytical correction was tested. The analysis of the simulated data was used to extract slope the theoretical correlating WM uptake and SUVRs values. The slopes were obtained for the different reference regions and then used to correct the dependency of SUVR with WM-SUVR_CGM_ over the patient data. Corrected SUVR values were obtained by applying a linear function that estimates the SUVR for the center of the previously estimated Gaussian (WM-SUVR_CGM_ = 1.79) using the measured SUVR_CGM_ and SUVR. The correlation between corrected SUVRs and WM-SUVR_CGM_ was assessed using Pearson’s correlation coefficients after the correction and compared with the original results.

## Results

### Patient data analysis

Of the 122 analyzed CN patients, 13 were discarded because of problems with PET/MR co-registering or MRI normalization or segmentation during the image analysis. Of the remaining, 27 (22%) were found amyloid-positive according to ADNI SUVR_WC_ threshold, and were also excluded from the subsequent analysis. On amyloid-negative patients, WM-SUVR_CGM_ ranged from 1.32 to 2.44, following a normal distribution with an average of 1.79 and a standard deviation of 0.24 (13.6%) (see Additional file 1: Supplementary Figure S3). Cerebellum WM, which is included inside the reference region for the calculation of SUVR_WC_, increased at a lower ratio (slope = 0.76, *R*^2^= 0.71) than the rest of the WM.

**Fig. 3:**
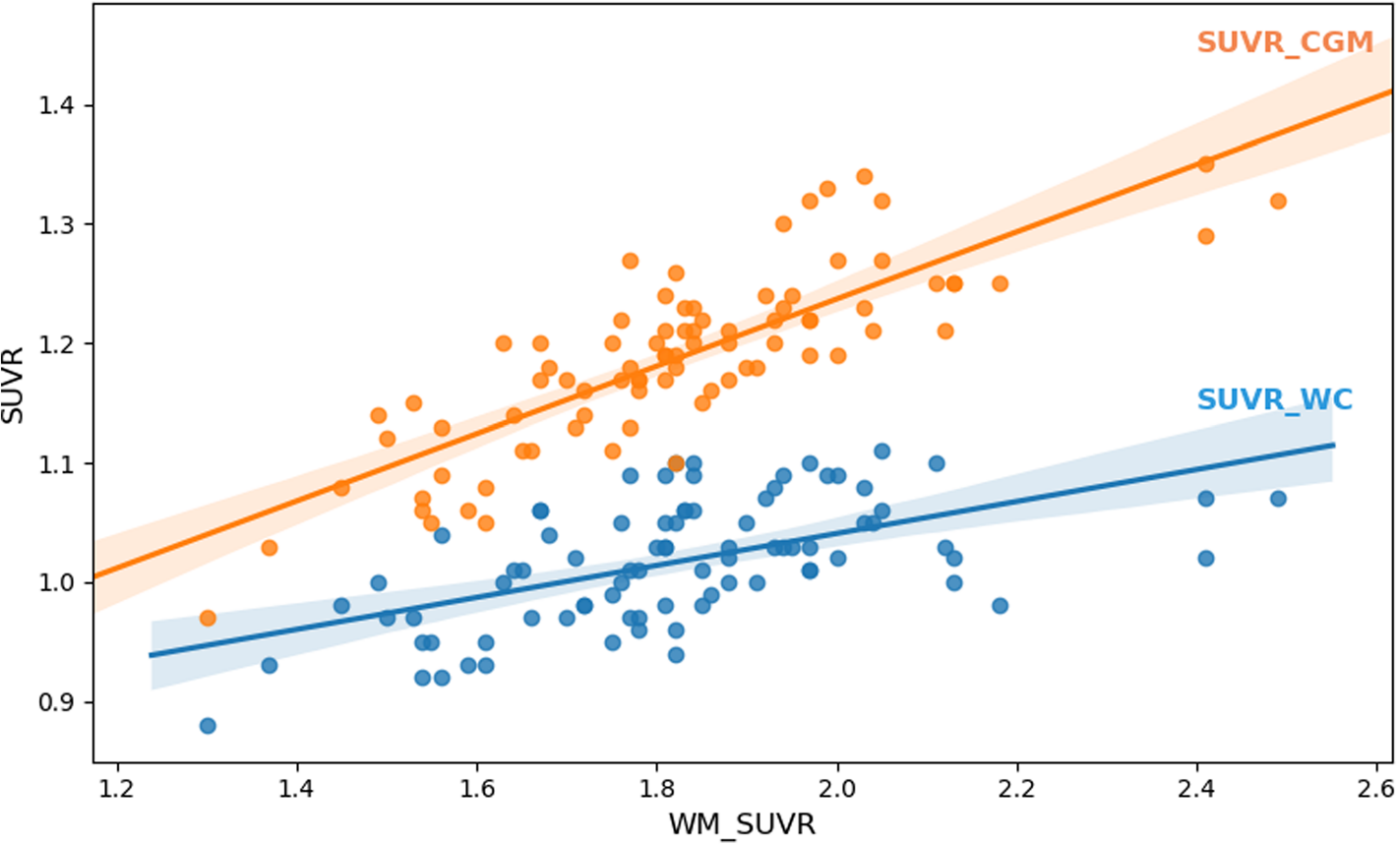
Relation between WM-SUVR_CGM_ and GM SUVRs. Orange dots (and orange line) are for the CGM as a reference region (SUVR_CGM_), while the blue dots (and blue line) are for the WC as a reference region (SUVR_WC_)

Figure 3 shows the relation of the measured SUVR_CGM_ (orange) and SUVR_WC_ (blue) with WM-SUVR_CGM_ on amyloid-negative patients. Both provided positive linear relations with Pearson’s coefficients of 0.82 (SUVR_CGM_ and WM-SUVR_CGM_) and 0.64 (SUVR_WC_ and WM-SUVR_CGM_). The increase of SUVR_CGM_ with WM-SUVR_CGM_ was more pronounced than that of SUVR_WC_ (slopes of 0.28 and 0.13, respectively). It is valuable to mention that no significant differences were found when using eroded WM, showing similar Pearson’s coefficients (0.80 and 0.61, respectively) and slopes (see Additional file 1: Supplementary Figure S4). Quantified values for each individual patient can be found at Additional file 1: Supplementary Table S2.

### Monte Carlo simulation

**Fig. 4:**
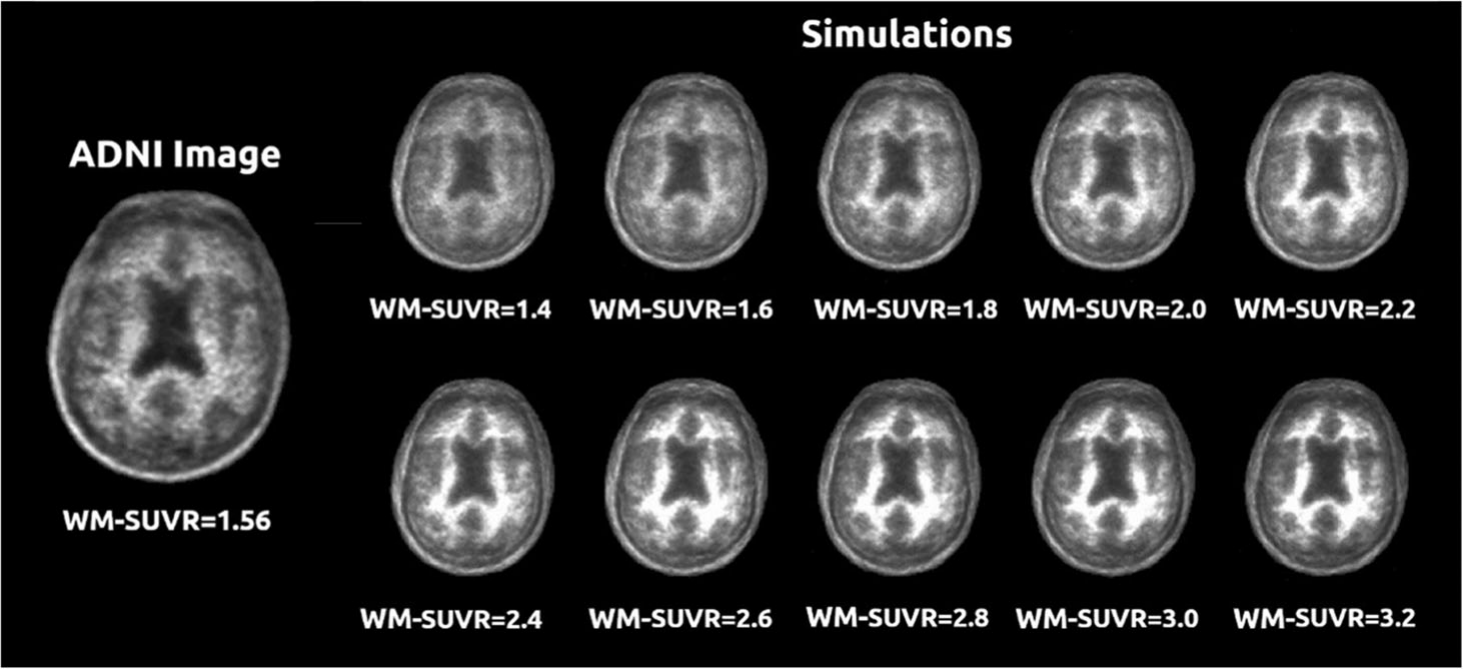
Simulations of the 10 activity maps with different WM uptakes and a fixed cortex uptake for one of the simulated patients

Realistic activity and attenuation maps were extracted for five ADNI patients from the previous cohort, with measured WM-SUVR_CGM_ of 1.56, 2.00, 2.12, 2.13, and 2.33. The resulting activity maps had theoretical SUVR_CGM_ of 0.96, 1.01, 1.11, 1.21, and 1.21. For each of the patients, WM was modified by multiplying the WM by a constant to produce maps with theoretical WM-SUVR_CGM_ values of 1.4, 1.6, 1.8, 2.0, 2.2, 2.4, 2.6, 2.8, 3.0, and 3.2, for each constant SUVR_CGM_ value. Simulated SUVR_WC_ were variable since the variation of WM-SUVR_CGM_ also affected the WM in the cerebellum. An example of the different simulations for one of the patients (ADNI RID 4579) can be observed in Fig. 4.

**Fig. 5:**
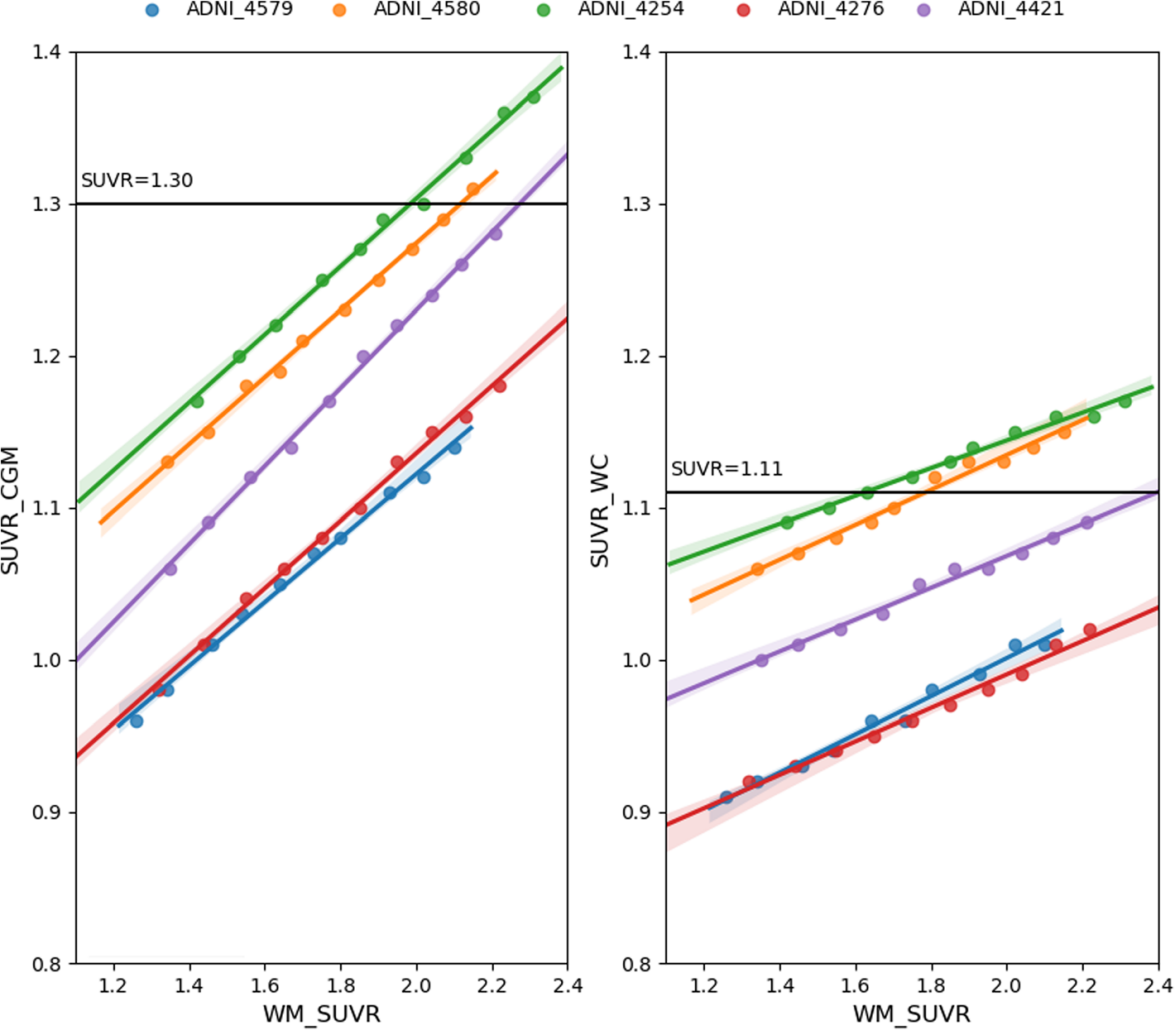
Relation between the measured SUVRs and WM-SUVR_CGM_. Each color represents one of the simulated patients. Values were measured for SUVR_CGM_ (left) and SUVR_WC_ (right). Threshold values of SUVR_CGM_ = 1.30 and SUVR_WC_ = 1.11 are plotted for representation purposes only

The simulated images were processed using the same steps detailed for patients. The measured relation between measured SUVR_CGM_, SUVR_WC_, and WM-SUVR_CGM_ on the simulations showed a slope of 0.226 ± 0.002 for the relation between SUVR_CGM_ and WM-SUVR_CGM_ and a slope of 0.108 ± 0.002 for the relation between SUVR_WC_ and WM-SUVR_CGM_. The results show that for the simulated SUVR ranges, there is not a dependency between the slope and the SUVR value, with estimates confidence levels compatible with zero. The results for the smoothed images can be observed in Fig. 5.

### Partial volume correction

**Fig. 6:**
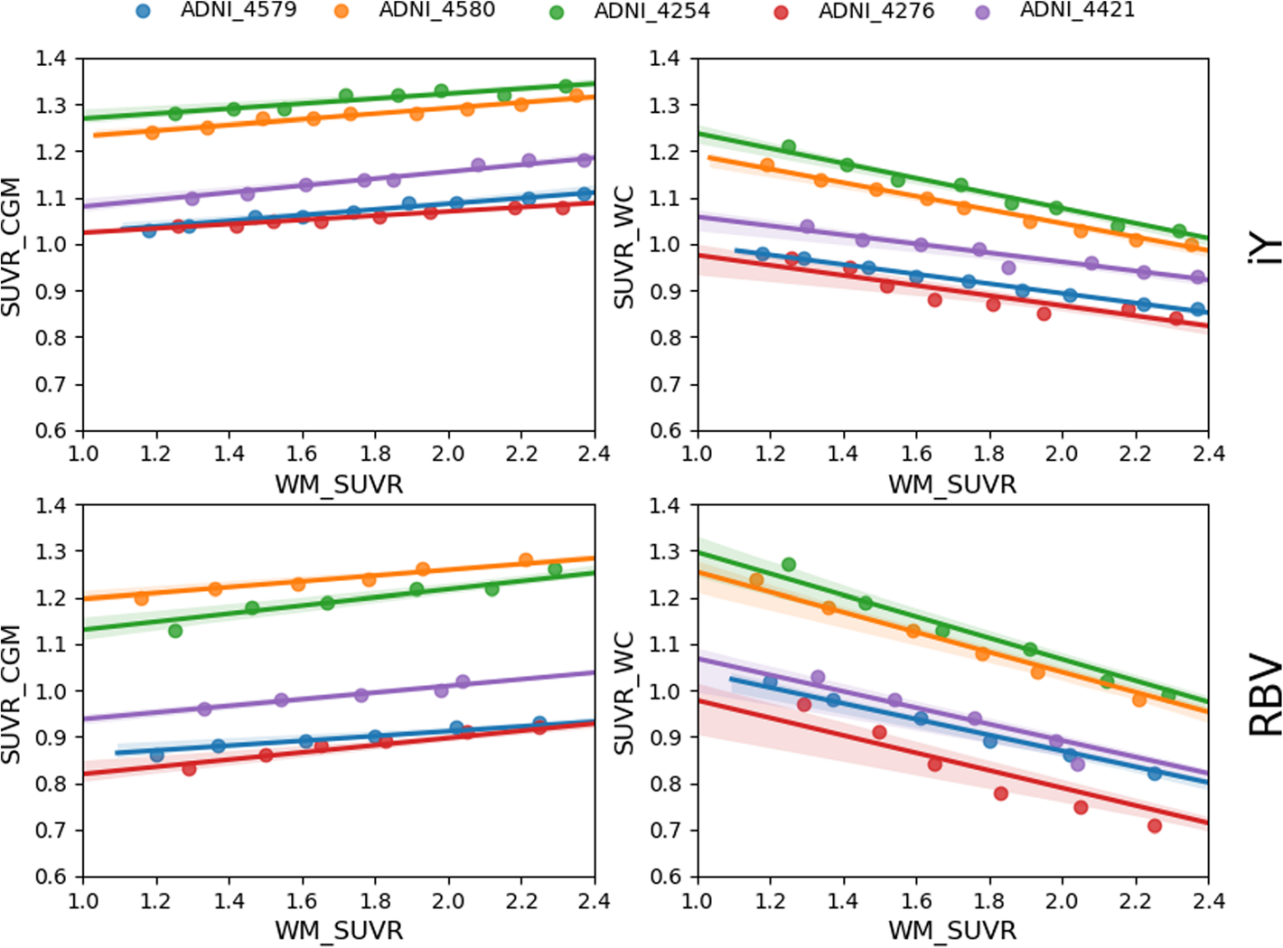
Relation between the measured SUVRs and WM-SUVR_CGM_ for PVC corrected images. Each color represents one of the simulated patients. Values are represented for SUVR_CGM_ (left) and SUVR_WC_ (right) and for iY (top) and RBV (bottom) PVCs

The ability of PVC to reduce the measured effect was evaluated by applying different PVC methods to the simulated data (examples of images processed by the different PVCs are provided in Additional file 1: Supplementary Figure S6). The results of the quantification for the PVC data are shown in Fig. 6. For the iY correction, the general linear model fitted to an average slope of 0.059 ± 0.002, while for the RBV the average slope was 0.070 ± 0.002, for SUVR_CGM_.

From the results, it is clear that applying PVC can reduce the spill-in, but it should be noted that for the two used methods, the intercept values for the different patient fits vary significantly. These variations between the different PVCs were patient-dependent, with iY providing a more accurate representation of the ground truth SUVR_CGM_ for low WM-SUVR_CGM_ (Additional file 1: Supplementary Figure S7 shows a comparison between the different measured values for RBV and iY and the ground truth simulated SUVR_CGM_). It is also important to point that we observed a reduction of SUVR_WC_ when increasing WM-SUVR_CGM_ when PVC is applied.

### Linear correction

Patient data was corrected by applying the linear slope extracted from the MC simulation. Corrected SUVR values were obtained by taking them to the average WM uptake, the center of the previously estimated Gaussian (WM-SUVR_CGM_ = 1.79). The same correction was applied to SUVR_WC_ by replacing with the corresponding slope:

$$ {\mathbf{SUVR}}_{\mathbf{CGM}-\mathbf{correc}}=\mathbf{0.226}\ast \left(\mathbf{1.79}-{\mathbf{WM}\mathrm{S}\mathbf{UVR}}_{\mathbf{CGM}}\right)+{\mathbf{SUVR}}_{\mathbf{CGM}} $$

$$ {\mathbf{SUVR}}_{\mathbf{WC}-\mathbf{correc}}=\mathbf{0.108}\ast \left(\mathbf{1.79}-{\mathbf{WMSUVR}}_{\mathbf{CGM}}\right)+{\mathbf{SUVR}}_{\mathbf{WC}} $$

**Fig. 7:**
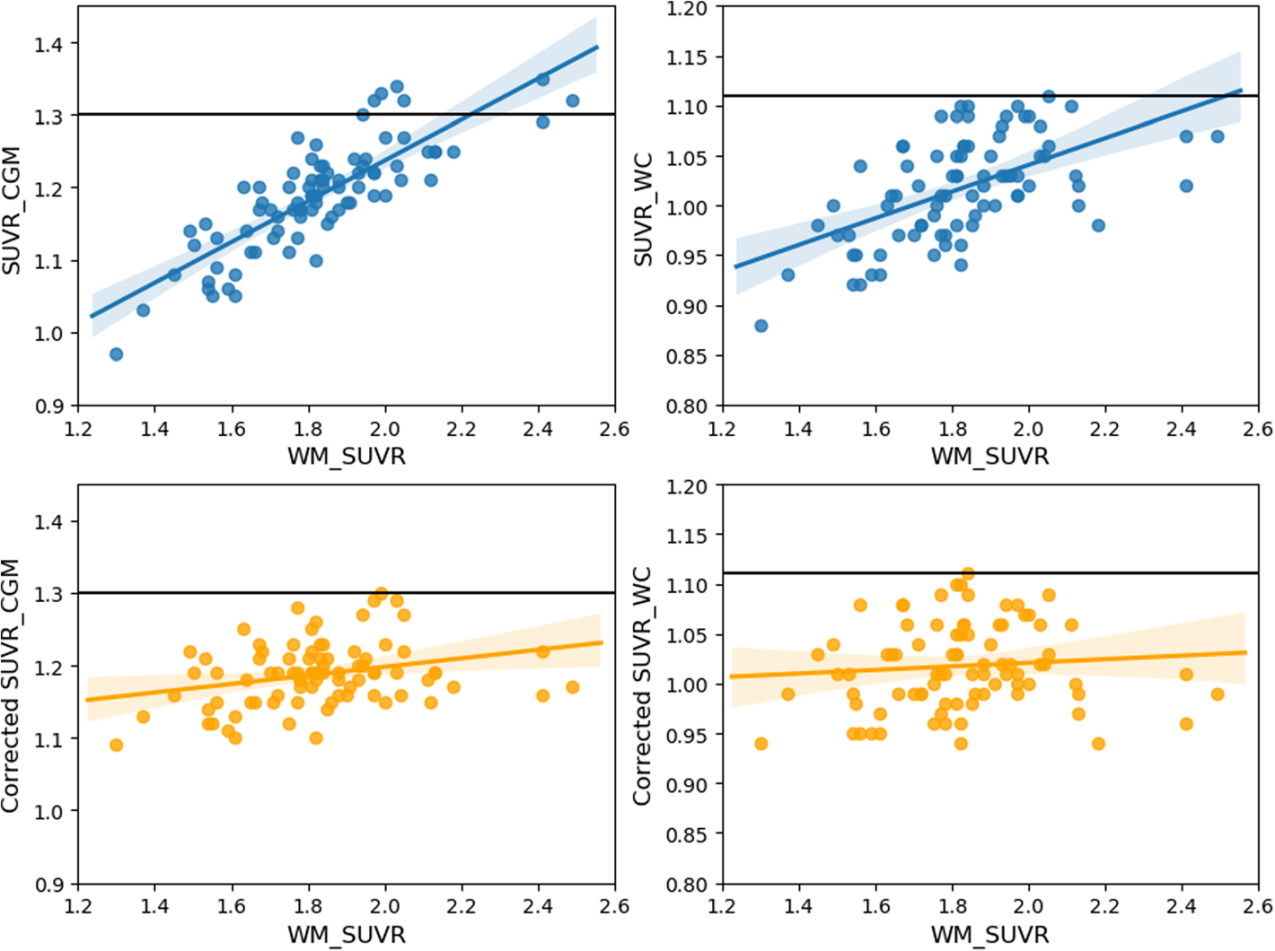
ADNI data corrected applying the linear relation obtained from Monte Carlo simulation. Blue points represent uncorrected data, while orange points represent corrected data. Values are represented for SUVR_CGM_ (left) and SUVR_WC_ (right). Threshold values of SUVR_CGM_ = 1.30 and SUVR_WC_ = 1.11 are plotted for representation purposes only

Pearson’s correlation coefficient between SUVR_CGM_ and WM-SUVR_CGM_ after the correction was ***r*** = 0.27. Pearson’s correlation coefficient between SUVR_WC_ and WM-SUVR_CGM_ after the correction was ***r*** = 0.18. The effect of this correction over our patient cohort is shown in Fig. 7.

## Discussion

Several publications have recently pointed to the fact that reference regions including WM provide better correlations with CSF levels and less longitudinal variability than the cerebellum cortex [11–14]. Some points in favor of WM reference regions could be that WM is a larger region potentially leading to less noise, more resistant to small degrees of misregistration and in the center of the scanner field-of-view. Nevertheless, WM uptake is known to be non-specific, and its mechanisms are largely unknown, so it is not an ideal candidate for a reference region. In this work, our main hypotheses was that an additional point to take into account is that including WM into the reference region will also compensate (at least partially), the spill-in of WM counts into the cortex, which would lead otherwise to artificially increased SUVR values, especially for healthy controls with low cortex uptake [26]. In order to assess this, we have investigated the variability of the WM uptake across a population of healthy patients, and then we used MC simulation to evaluate, first, if these changes are enough to produce significant variations in cortex SUVR values, and second, if a reference region including WM (in our case the WC (GM+WM) effectively minimizes these variations.

WM values on the evaluated patient cohort were in the range of 1.32–2.44, following a normal distribution centered in 1.79 with a percentage standard deviation of 13.6%. These values are in good agreement with previously published results [51] that reported WM averages of 1.92 ± 0.23 for healthy patients. We also observed that the increase of uptake in the cerebellum WM was slower than in the rest of the WM (ratio approximately of ≈ 0.8). This difference could be attributed to PVE itself, as the size of the cerebellum WM is relatively small when compared to the rest of the WM. We observed a positive correlation between measured SUVR values and WM uptake, which was more prominent when using the CGM (*r* = 0.82) as a reference region, than when using a reference region containing WM, as the WC (*r* = 0.64). We observed that SUVR_CGM_ increased by 0.28 units when WM-SUVR_CGM_ increases one unit, while SUVR_WC_ increased 0.13 units when WM-SUVR_CGM_ increased one unit. This correlation is significantly higher than previously reported in ^11^C-PIB [27]. This can be related to the fact that WM retention in ^18^F-based tracers is significantly higher than in ^11^C-PIB [52]. In addition, in the cited publication, authors use both amyloid-positive and amyloid-negative CN patients, which would also reduce the observed correlation (see Additional file 1: Supplementary Figure S5). We also showed that the correlation is present independently of using all the WM or only the eroded WM as defined by ADNI [16], pointing that the spill-out of cortex counts into the WM is irrelevant in comparison with the WM spill-in. This is explained, again, by the fact that we are evaluating only amyloid-negative patients, where WM uptake is significantly higher than cortex uptake.

Regarding our MC experiments, we simulated realistic maps with fixed cortex uptake, and variable WM uptake, and evaluated the variations of measured SUVRs following the same methodology used in patients. This way we isolate WM spill-in from any other physical or physiological effects, allowing a precise investigation of this particular effect. These experiments showed that the introduced WM variability produced correlations similar to those observed in patients. SUVR_CGM_ increased by 0.23 units when WM-SUVR_CGM_ increases one unit, while SUVR_WC_ increased 0.11 units when WM-SUVR_CGM_ increased one unit.

To evaluate the ability of PVC methods to remove this effect, we tested two different methods, iY and RBV, over the simulated data. These methods were used instead of more widespread alternatives such as Müller-Gärtner (MG) and GTM, since they have the ability to produce a corrected image, enabling to reproduce the exact same processing pipeline (with the removal of the smoothing to 8 mm) used in patient data. Both methods were able to reduce the observed dependency, reducing the aforementioned 0.23 slope for SUVR_CGM_ to 0.06 and 0.07, respectively. It is important to remark that corrected SUVR for the different PVC methods were qualitatively different, something that has been previously highlighted in other publications [53, 54]. Once PVE was corrected, using the WC as the reference region produced a reduction of the SUVRs as WM increased. On the one hand, this finding reinforces our hypothesis of the compensation of spill-in counts by including WM counts as a key to the good performance of these reference regions. On the other hand, it seems to contradict previous findings pointing to the combination of WM reference regions and PVC as optimal for longitudinal reliability of the measurements and threshold-based separation [15, 55], but this is not necessarily the case. In both cases, both amyloid-negative and positive subjects compose the patient cohort, and WM emerges as the best-suited reference region based on longitudinal performance. In such a case of study, spill-out due to atrophy and cortical thickness variations might play a more prominent role than spill-in, since reduced WM-GM contrast is expected. Furthermore, WM variability has been shown to influence the accuracy of MG-based PVCs in cortical GM and also CGM, while RBV and iY account for within-compartment variability [25], so our findings might still be compatible with those in these publications.

In brief, our results suggest that the correlation observed in patient data is largely produced by WM spill-in, and that it is reduced when introducing WM into the reference region. This could be an explanation as to why reference regions including WM perform better than CGM in longitudinal studies [13–15]. In addition, we observed that a very similar reduction of the effect of the PVE could be obtained by applying PVC and using CGM as a reference region, as it can be observed in Fig. 6. This later implementation will be more convenient, as CGM is, theoretically, a better reference region. Nevertheless, further work would be required to investigate the effect of iY, RBV and other PVC methodologies on the analysis of longitudinal data.

Finally, we evaluated the performance of a simple analytical correction, by applying the observed slope obtained in our MC simulation (that is strictly derived from PVE) to the patient data, in order to remove the previously described dependency. The results are presented in Fig. 7. This simple correction was effective in reducing the previously observed correlation coefficients between SUVR_CGM_ and WM-SUVR_CGM_ (from 0.82 to 0.27) and between SUVR_WC_ and WM-SUVR_CGM_ (from 0.64 to 0.18). It is important to remark that, as it can be observed on Fig. 7 (bottom), applying our linear correction does not remove all the correlation. This could be explained by the differences in resolution between our MC model and the real PET scanner (5–10%), or by other factors correlating SUVR_CGM_ and WM-SUVR_CGM_ that were not taken into account on our MC simulation. The main limitation of the presented correction is that the presented formulas will be applicable only for the ADNI cohort, or for other studies that have adopted the ADNI processing methodology, and only for amyloid-negative patients. Nevertheless, this type of correction might be of interest since it does not require additional processing of the image and it can be applied directly to previously calculated data tables and SUVR values, such as those provided by ADNI PET processing core at Berkeley. For applying this correction to cohorts with different image processing pipelines, the simulation needs to be repeated in order to recalculate the slopes and average WM-SUVR_CGM_ for the proposed formulas. About the application on amyloid-positive patients, a clear limitation of the proposed methodology is that high cortex uptake will change the contrast relations between GM and WM, and thus, the amount of PVE and the calculated slope might also change [23]. A much more complex analysis including simulations for amyloid-positive patients in the whole range of SUVRs would be needed to generalize the proposed correction to the entire cohort. Nevertheless, the correlation observed in Fig. 3 is expected to decrease when the cortical amyloid load is high.

About some additional limitations of the present work, our activity maps were extracted only for 5 of the initial cohort of 82 patients, all of them were scanned with the GE Discovery STE scanner. Applying the derived formulas to the entire cohort, we are assuming, first, that the smoothing values proposed by ADNI (see Additional file 1: Supplementary Table S1) correctly harmonize the resolution of the scanners in the cohort, and second, that the range of simulated ground truth SUVR values (0.9–1.21) is representative of the values on the cohort. Future work could expand the presented simulations by including different scanners and more ground truth SUVRs.

## Conclusions

We have observed that there is a significant positive correlation between measured SUVRs and WM uptake in amyloid-negative patients, and that this correlation is reduced by using reference regions including WM. This could be an explanation for the good performance of reference regions including WM. By using MC simulation, we demonstrated that this correlation is largely produced by PVE, and that it can be removed by using PVC. These results shall be of special interest for situations where primarily healthy populations are used, such as the calculation of SUVR positivity thresholds. We have proposed a correction that can be applied directly to previously calculated SUVR values in such cases.

## Supplementary information

**Additional file 1:** **Figure S1.** Detailed view of some of the results of the image processing and their relationships. On the top: Original MRI and PET images and results of the MRI normalization (warped atlas) and segmentation. On the bottom: GM patient-specific atlas generated from the warped atlas and the GM. PVC labels including WM and CSF together with the GM specific atlas and BrainVIset input (iteration 0) activity and attenuation maps. **Figure S2.** Comparison of SUVR values measured on our lab (x-axis) and data calculated by the ADNI PET Core at Berkeley (y-axis) for our final subject sample. Average differences between Berkeley calculations and ours were found to be ± 4.6%. These small differences are mainly due to different processing pipelines, such as different segmentation methods (CAT vs. Freesurfer), atlas (Hammersmith vs Desikan) or quantification space (patient vs. MNI). **Figure S3.** Distribution of WM values across the studied ADNI2 sub-sample. Only amyloid-negative patients are presented. The bars represent the number of patients on each bin of the histogram, while the black solid line represent the Gaussian distribution of the histogram. **Figure S4.** Visual comparison of the correlations of cortex SUVR (x-axis) with WM-SUVRCGM, using both the whole WM (green) and the Eroded WM (purple) as WM regions. **Figure S5.** Relation between WM-SUVRCGM (x-axis) and GM SUVR using the whole cerebellum as a reference region (SUVRWC). The Figure represents the actual cohort used for this work (amyloid-negative patients, blue points), and patients excluded for being positive according to the ADNI SUVRWC =1.11 threshold (red and orange dots). The correlation coefficient between WM-SUVRCGM and SUVRWC when including amyloid-positive patients was r=0.55. **Figure 6.** Example images for the different levels of processing for some of the simulated images. Each row shows the original image (left), smoothed image (center-left), atlas used for the PVC (center). RBV-corrected image (center-right) and iY-corrected image (right), for each of the cases. **Figure 7.** Comparison of ground truth and measured SUVRCGM values for RBV (blue lines). **Table S1.** Measured PF for each of the scanners present in the ADNI database (measured by the ADNI) and smoothing applied to each of the scanners to obtain an isotropic 8-mm resolution (as proposed by the ADNI). **Table 2.** Quantification results for all the analyzed patients, including the ADNI label for the patient (Patient), the quantified Cortex average (Cortex AVG), cerebellum grey matter average (CGM AVG), whole cerebellum average (WC AVG), white matter average (WM AVG), SUVRCWM, SUVRWC and the PET scanner.

## Data Availability

Patient data used in the preparation of this article were obtained from the Alzheimer’s Disease Neuroimaging Initiative (ADNI) database. Data from the ADNI is open and can be obtained from the ADNI data repositories [30]. In this work, we included 122 cognitively normal (CN) patients recruited at the start of ADNI2 who underwent a baseline structural MRI and ^18^F-AV-45 (florbetapir) PET scan. The details of the data downloaded from ADNI, in addition to the quantification values obtained from these data during the execution of this work, can be found at Additional file 1: Supplementary Table S2. The BrainViset derived activity and attenuation maps are also available for sharing. For acquiring these materials, please contact Jesús Silva-Rodríguez (jesus@qubiotech.com) or Pablo Aguiar (pablo.aguiar.fernandez@sergas.es)
