# Supplementary material for: Spill-in counts in the quantification of 18F-florbetapir on Aβ-negative subjects: the effect of including white matter in the reference region
Source: EJNMMI Phys. 2019 Dec 19;6:27. doi: 10.1186/s40658-019-0258-7 (PMC6923310; doi:10.1186/s40658-019-0258-7)
Supplement: Supplementary file 1 — Additional file 1: Figure S1. Detailed view of some of the results of the image processing and their relationships. On the top: Original MRI and PET images and results of the MRI normalization (warped atlas) and segmentation. On the bottom: GM patient-specific atlas generated from the warped atlas and the GM. PVC labels including WM and CSF together with the GM specific atlas and BrainVIset input (iteration 0) activity and attenuation maps. Figure S2. Comparison of SUVR values measured on our lab (x-axis) and data calculated by the ADNI PET Core at Berkeley (y-axis) for our final subject sample. Average differences between Berkeley calculations and ours were found to be ± 4.6%. These small differences are mainly due to different processing pipelines, such as different segmentation methods (CAT vs. Freesurfer), atlas (Hammersmith vs Desikan) or quantification space (patient vs. MNI). Figure S3. Distribution of WM values across the studied ADNI2 sub-sample. Only amyloid-negative patients are presented. The bars represent the number of patients on each bin of the histogram, while the black solid line represent the Gaussian distribution of the histogram. Figure S4. Visual comparison of the correlations of cortex SUVR (x-axis) with WM-SUVRCGM, using both the whole WM (green) and the Eroded WM (purple) as WM regions. Figure S5. Relation between WM-SUVRCGM (x-axis) and GM SUVR using the whole cerebellum as a reference region (SUVRWC). The Figure represents the actual cohort used for this work (amyloid-negative patients, blue points), and patients excluded for being positive according to the ADNI SUVRWC =1.11 threshold (red and orange dots). The correlation coefficient between WM-SUVRCGM and SUVRWC when including amyloid-positive patients was r=0.55. Figure 6. Example images for the different levels of processing for some of the simulated images. Each row shows the original image (left), smoothed image (center-left), atlas used for the PVC (center). RBV-corrected image (cen [file 40658_2019_258_MOESM1_ESM.docx]

**Supplementary Data**

**Supplementary Figure 1:** Detailed view of some of the results of the image processing and their relationships. On the top: Original MRI and PET images and results of the MRI normalization (warped atlas) and segmentation. On the bottom: GM patient-specific atlas generated from the warped atlas and the GM. PVC labels including WM and CSF together with the GM specific atlas and BrainVIset input (iteration 0) activity and attenuation maps.


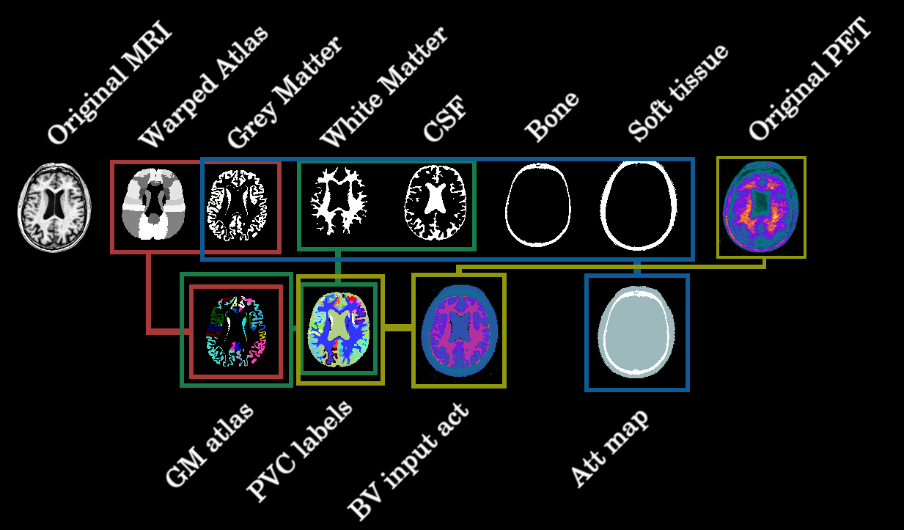


**
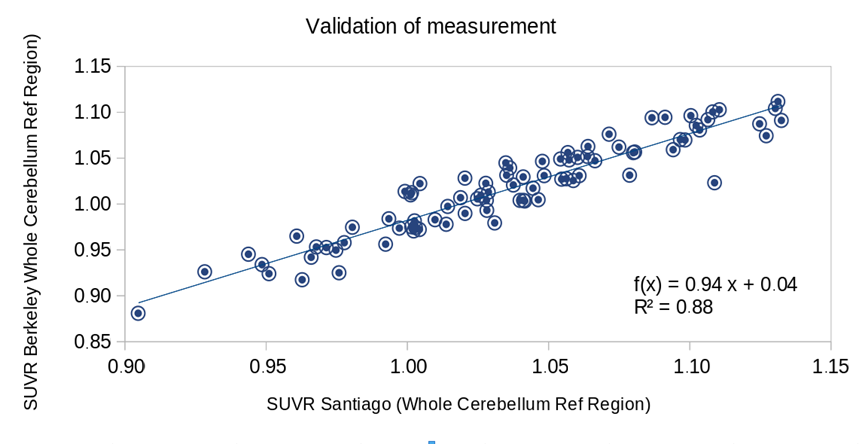
Supplementary Figure 2:** Comparison of SUVR values measured on our lab (x-axis) and data calculated by the ADNI PET Core at Berkeley (y-axis) for our final subject sample. Average differences between Berkeley calculations and ours were found to be ± 4.6%. These small differences are mainly due to different processing pipelines, such as different segmentation methods (CAT vs. Freesurfer), atlas (Hammersmith vs. Desikan) or quantification space (patient vs. MNI).

**
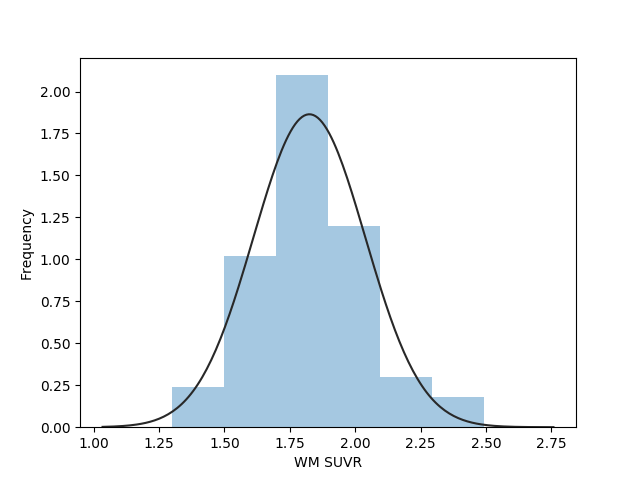
Supplementary Figure 3:** Distribution of WM values across the studied ADNI2 sub-sample. Only amyloid-negative patients are presented. The bars represent the number of patients on each bin of the histogram, while the black solid line represent the Gaussian distribution of the histogram.

**
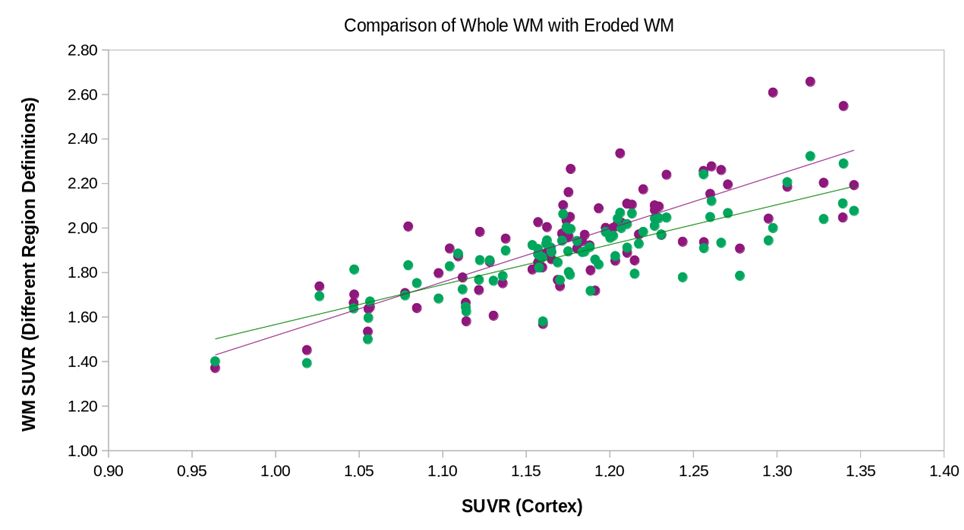
8iu7Supplementary Figure 4:** Visual comparison of the correlations of cortex SUVR (x-axis) with WM-SUVR_CGM_, using both the whole WM (green) and the Eroded WM (purple) as WM regions.

**Supplementary Figure 5:** Relation between WM-SUVR_CGM_ (x-axis) and GM SUVR using the whole cerebellum as a reference region (SUVR_WC_). The Figure represents the actual cohort used for this work (amyloid-negative patients, blue points), and patients excluded for being positive according to the ADNI SUVR_WC_ =1.11 threshold (red and orange dots). The correlation coefficient between WM-SUVR_CGM_ and SUVR_WC_ when including amyloid-positive patients was r=0.55

**
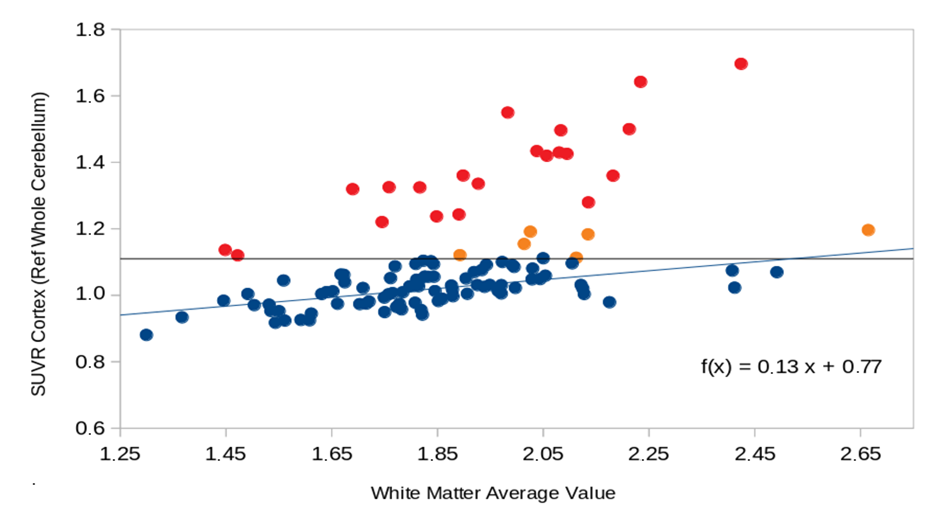
**


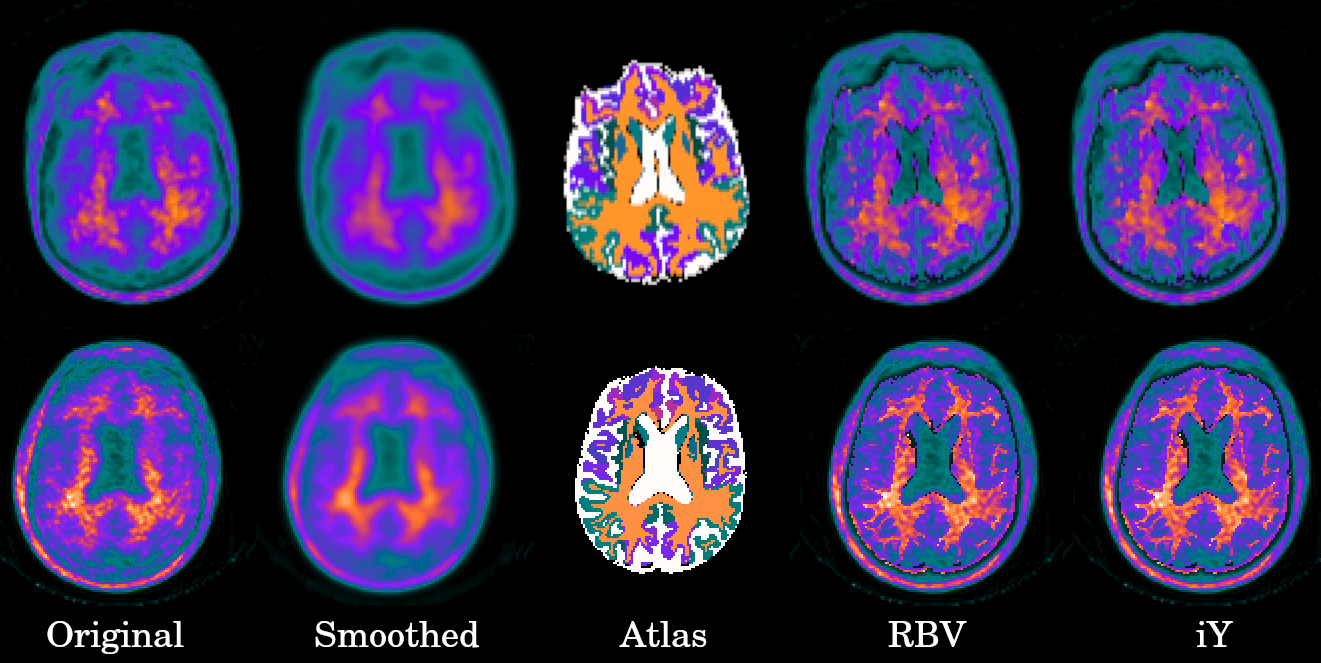
**Supplementary Figure 6:** Example images for the different levels of processing for some of the simulated images. Each row shows the original image (left), smoothed image (center-left), atlas used for the PVC (center). RBV-corrected image (center-right) and iY-corrected image (right), for each of the cases.

**
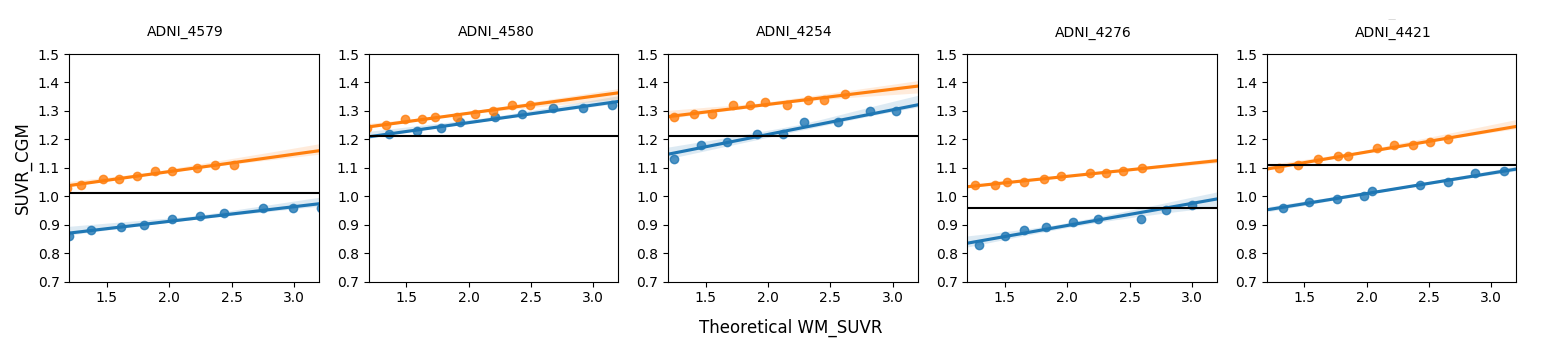
Supplementary Figure 7:** Comparison of ground truth and measured SUVR_CGM_ values for RBV (blue lines) and iY (orange line) for different WM- SUVR_CGM_.

**Supplementary Table 1:** Measured PF for each of the scanners present in the ADNI database (measured by the ADNI) and smoothing applied to each of the scanners to obtain an isotropic 8-mm resolution (as proposed by the ADNI).

|  |  | **Measured PSF** | | **Applied Smoothing** | |
| --- | --- | --- | --- | --- | --- |
| **Manufacturer** | **Scanner** | **in-plane** | **axial** | **XY smoothing** | **Z smoothing** |
| Siemens | HRRT | 4.5 | 4.5 | 6.0 | 6.0 |
| Siemens | BioGraph 1080 | 5.5 | 5.5 | 5.5 | 5.5 |
| Siemens | BioGraph TruePoint (1093/1094) | 5.5 | 5.5 | 5.5 | 5.5 |
| Siemens | BioGraph mCT | 5.5 | 5.5 | 5.5 | 5.5 |
| Philips | GemTF Sharp | 5.5 | 5.5 | 4.5 | 5.0 |
| Siemens | ECAT Exact HR+ | 6.0 | 6.0 | 5.0 | 5.0 |
| Siemens | ECAT Exact HR | 5.5 | 7.0 | 5.5 | 3.5 |
| GE | Disc600_3D IR | 5.5 | 6.0 | 5.5 | 5.0 |
| GE | Disc600_RP | 6.0 | 7.0 | 5.0 | 3.5 |
| GE | Disc690_3D IR | 5.5 | 6.0 | 5.5 | 5.0 |
| GE | Disc690_RP | 6.0 | 7.0 | 5.0 | 3.5 |
| GE | DiscRX_3D IR | 5.5 | 6.0 | 5.5 | 5.0 |
| GE | DiscRX RP | 6.0 | 7.0 | 5.0 | 3.5 |
| GE | DiscSTE_3D IR | 5.5 | 6.0 | 5.5 | 5.0 |
| GE | DiscSTE_RP | 6.0 | 7.0 | 5.0 | 3.5 |
| GE | DiscST_3D IR | 6.0 | 6.0 | 5.0 | 5.0 |
| GE | DiscST_FORE 2D IR | 7.5 | 7.0 | 3.0 | 3.5 |
| GE | DiscST_RP | 7.0 | 7.0 | 3.5 | 3.5 |
| GE | DiscLS_FORE 2D IR | 6.5 | 7.0 | 4.5 | 3.0 |
| GE | DiscLS_RP | 6.0 | 7.5 | 5.0 | 2.0 |
| GE | Advance_FORE 2D IR | 6.5 | 7.0 | 4.5 | 3.0 |
| GE | Advance_RP | 6.0 | 7.5 | 5.0 | 2.0 |
| Philips | Allegro | 7.5 | 7.5 | 3.0 | 3.0 |
| Philips | Gemini GXL | 7.5 | 7.5 | 3.0 | 3.0 |
| Philips | Gemini | 7.5 | 7.5 | 3.0 | 3.0 |
| Siemens | BioGraph (47 slice) | 7.5 | 7.0 | 2.0 | 3.0 |
| Siemens | ECAT Accel | 7.5 | 7.0 | 2.0 | 3.0 |
| Siemens | ECAT Exact | 7.5 | 7.0 | 2.0 | 3.0 |

**Supplementary Table 2:** Quantification results for all the analyzed patients, including the ADNI label for the patient (Patient), the quantified Cortex average (Cortex AVG), cerebellum grey matter average (CGM AVG), whole cerebellum average (WC AVG), white matter average (WM AVG), SUVR_CWM_, SUVR_WC_ and the PET scanner.

| Patient | Cortex AVG | CGM AVG | WC AVG | WM AVG | SUVR_CWM_ | SUVR_WC_ | Scanner |
| --- | --- | --- | --- | --- | --- | --- | --- |
| 002_S_4225 | 1.76 | 1.01 | 1.17 | 2.08 | 1.74 | 1.51 | GEMINI TF TOF |
| 002_S_4262 | 1.75 | 1.03 | 1.14 | 1.98 | 1.71 | 1.54 | GEMINI TF TOF |
| 002_S_4270 | 2.00 | 1.07 | 1.22 | 2.23 | 1.87 | 1.64 | GEMINI TF TOF |
| 003_S_4119 | 1.31 | 1.04 | 1.26 | 2.13 | 1.25 | 1.04 | SIEMENS 1094 |
| 003_S_4288 | 1.49 | 1.09 | 1.27 | 2.01 | 1.36 | 1.18 | SIEMENS 1094 |
| 003_S_4555 | 1.38 | 1.12 | 1.24 | 1.84 | 1.23 | 1.11 | SIEMENS 1094 |
| 003_S_4644 | 1.29 | 1.01 | 1.13 | 1.89 | 1.28 | 1.14 | SIEMENS 1094 |
| 003_S_4872 | 1.29 | 1.07 | 1.20 | 1.83 | 1.21 | 1.08 | SIEMENS 1094 |
| 003_S_4900 | 1.24 | 1.03 | 1.16 | 1.93 | 1.20 | 1.07 | SIEMENS 1094 |
| 006_S_4150 | 1.17 | 1.00 | 1.16 | 1.81 | 1.17 | 1.01 | Siemens HR+ |
| 007_S_4387 | 1.27 | 1.04 | 1.24 | 1.97 | 1.22 | 1.03 | Discovery RX |
| 007_S_4488 | 1.22 | 1.00 | 1.15 | 1.93 | 1.22 | 1.06 | Discovery RX |
| 007_S_4516 | 1.22 | 1.01 | 1.17 | 1.88 | 1.21 | 1.05 | Discovery RX |
| 007_S_4620 | 1.12 | 1.00 | 1.09 | 1.56 | 1.13 | 1.04 | Discovery RX |
| 007_S_4637 | 1.30 | 1.03 | 1.15 | 1.82 | 1.26 | 1.13 | Discovery RX |
| 009_S_4388 | 1.07 | 0.95 | 1.07 | 1.50 | 1.12 | 1.00 | Discovery LS |
| 009_S_4612 | 1.21 | 1.02 | 1.11 | 1.47 | 1.19 | 1.09 | Discovery LS |
| 010_S_4345 | 1.15 | 0.98 | 1.14 | 1.78 | 1.17 | 1.00 | Siemens HR+ |
| 011_S_4105 | 1.21 | 1.03 | 1.16 | 1.76 | 1.17 | 1.04 | SIEMENS 1094 |
| 011_S_4120 | 1.55 | 1.06 | 1.18 | 1.82 | 1.47 | 1.32 | SIEMENS 1094 |
| 011_S_4222 | 1.13 | 1.02 | 1.13 | 1.65 | 1.11 | 1.00 | SIEMENS 1094 |
| 011_S_4278 | 1.75 | 1.06 | 1.24 | 2.10 | 1.65 | 1.41 | SIEMENS 1094 |
| 012_S_4545 | 1.31 | 1.07 | 1.24 | 2.03 | 1.23 | 1.06 | Discovery LS |
| 013_S_4579 | 0.94 | 0.97 | 1.04 | 1.30 | 0.97 | 0.91 | Discovery STE |
| 013_S_4580 | 1.22 | 1.02 | 1.19 | 1.81 | 1.19 | 1.02 | Discovery STE |
| 013_S_4616 | 1.72 | 1.04 | 1.22 | 2.18 | 1.65 | 1.41 | Discovery STE |
| 014_S_4080 | 1.31 | 1.04 | 1.16 | 1.77 | 1.27 | 1.13 | Philips Medical Systems |
| 014_S_4093 | 1.49 | 1.07 | 1.27 | 2.11 | 1.39 | 1.18 | Philips Medical Systems |
| 016_S_4121 | 1.14 | 1.03 | 1.17 | 1.75 | 1.11 | 0.98 | BioGraph TruePoint |
| 016_S_4638 | 1.36 | 1.07 | 1.24 | 2.05 | 1.27 | 1.09 | BioGraph TruePoint |
| 016_S_4688 | 1.60 | 1.10 | 1.24 | 2.14 | 1.45 | 1.29 | BioGraph TruePoint |
| 016_S_4952 | 1.21 | 1.05 | 1.20 | 1.85 | 1.15 | 1.01 | BioGraph TruePoint |
| 018_S_4349 | 1.24 | 1.02 | 1.17 | 1.76 | 1.22 | 1.06 | GEMS Advance |
| 018_S_4399 | 1.30 | 1.10 | 1.23 | 1.90 | 1.18 | 1.06 | GEMS Advance |
| 018_S_4400 | 1.70 | 1.06 | 1.20 | 2.04 | 1.61 | 1.42 | GEMS Advance |
| 019_S_4367 | 1.25 | 1.02 | 1.21 | 1.85 | 1.22 | 1.03 | BioGraph TruePoint |
| 019_S_4835 | 1.34 | 1.01 | 1.21 | 1.97 | 1.32 | 1.11 | BioGraph TruePoint |
| 021_S_4254 | 1.36 | 1.12 | 1.31 | 2.12 | 1.21 | 1.04 | Discovery STE |
| 021_S_4276 | 1.00 | 0.94 | 1.08 | 1.59 | 1.06 | 0.93 | Discovery STE |
| 021_S_4335 | 1.42 | 1.01 | 1.15 | 1.89 | 1.41 | 1.23 | Discovery STE |
| 021_S_4421 | 1.13 | 0.99 | 1.12 | 1.72 | 1.14 | 1.00 | Discovery STE |
| 022_S_4173 | 1.20 | 0.96 | 1.10 | 1.81 | 1.24 | 1.09 | GEMINI TF TOF |
| 022_S_4196 | 1.32 | 1.00 | 1.12 | 1.85 | 1.32 | 1.18 | GEMINI TF TOF |
| 022_S_4266 | 1.74 | 1.05 | 1.20 | 2.08 | 1.65 | 1.44 | GEMINI TF TOF |
| 022_S_4291 | 1.57 | 1.03 | 1.15 | 1.90 | 1.52 | 1.36 | GEMINI TF TOF |
| 023_S_4020 | 1.28 | 1.05 | 1.22 | 1.97 | 1.22 | 1.05 | SIEMENS 1093 |
| 023_S_4164 | 1.20 | 1.02 | 1.21 | 1.82 | 1.18 | 0.99 | SIEMENS 1093 |
| Patient | **Cortex AVG** | **CGM AVG** | **WC AVG** | **WM AVG** | **SUVR_CWM_** | **SUVR_WC_** | **Scanner** |
| 023_S_4448 | 1.26 | 1.01 | 1.15 | 1.92 | 1.24 | 1.10 | SIEMENS 1093 |
| 024_S_4084 | 1.16 | 1.00 | 1.13 | 1.78 | 1.16 | 1.03 | Siemens HR+ |
| 029_S_4384 | 1.18 | 1.01 | 1.11 | 1.67 | 1.17 | 1.06 | Siemens HRRT |
| 029_S_4385 | 1.46 | 1.07 | 1.13 | 1.69 | 1.36 | 1.29 | Siemens HRRT |
| 029_S_4585 | 1.21 | 1.01 | 1.12 | 1.84 | 1.20 | 1.09 | Siemens HRRT |
| 029_S_4652 | 1.21 | 1.04 | 1.10 | 1.45 | 1.17 | 1.10 | Siemens HRRT |
| 031_S_4021 | 1.18 | 1.01 | 1.21 | 1.78 | 1.17 | 0.98 | Siemens HR+ |
| 031_S_4474 | 1.51 | 1.01 | 1.12 | 1.76 | 1.50 | 1.35 | Siemens HR+ |
| 031_S_4496 | 1.24 | 1.01 | 1.17 | 1.94 | 1.23 | 1.06 | Siemens HR+ |
| 032_S_4348 | 1.60 | 1.07 | 1.18 | 2.06 | 1.50 | 1.36 | Siemens HRRT |
| 032_S_4386 | 2.75 | 1.19 | 1.42 | 3.35 | 2.30 | 1.93 | Siemens HRRT |
| 032_S_4429 | 1.48 | 1.07 | 1.21 | 2.14 | 1.39 | 1.22 | Siemens HRRT |
| 032_S_4921 | 1.40 | 1.05 | 1.27 | 2.03 | 1.34 | 1.10 | Siemens HRRT |
| 033_S_4176 | 1.39 | 1.02 | 1.14 | 1.75 | 1.36 | 1.22 | Discovery ST |
| 033_S_4177 | 1.59 | 1.23 | 1.43 | 2.41 | 1.29 | 1.11 | Discovery ST |
| 033_S_4508 | 1.53 | 1.01 | 1.15 | 1.93 | 1.52 | 1.33 | Discovery ST |
| 035_S_4082 | 1.40 | 1.12 | 1.36 | 2.18 | 1.25 | 1.03 | SIEMENS 1094 |
| 035_S_4464 | 1.22 | 1.01 | 1.15 | 1.84 | 1.21 | 1.06 | SIEMENS 1094 |
| 036_S_4491 | 1.04 | 0.98 | 1.08 | 1.54 | 1.06 | 0.96 | Siemens HR+ |
| 036_S_4878 | 1.27 | 1.02 | 1.18 | 1.95 | 1.24 | 1.08 | Siemens HR+ |
| 037_S_4308 | 1.21 | 1.00 | 1.13 | 1.81 | 1.21 | 1.07 | Siemens HR+ |
| 037_S_4410 | 1.07 | 0.99 | 1.08 | 1.45 | 1.08 | 0.99 | Siemens HR+ |
| 041_S_4200 | 1.23 | 1.03 | 1.15 | 1.67 | 1.20 | 1.08 | Siemens HR+ |
| 041_S_4427 | 1.12 | 0.93 | 1.07 | 1.63 | 1.20 | 1.04 | Siemens HR+ |
| 068_S_4174 | 1.78 | 1.08 | 1.22 | 2.21 | 1.65 | 1.46 | Discovery ST |
| 070_S_5040 | 1.32 | 1.11 | 1.32 | 1.97 | 1.19 | 1.00 | GEMS Advance |
| 072_S_4103 | 1.22 | 1.02 | 1.15 | 1.81 | 1.19 | 1.06 | BioGraph TruePoint |
| 072_S_4391 | 1.27 | 1.07 | 1.24 | 2.00 | 1.19 | 1.03 | BioGraph TruePoint |
| 073_S_4155 | 2.04 | 1.07 | 1.23 | 2.42 | 1.91 | 1.66 | Discovery STE |
| 073_S_4382 | 1.17 | 0.98 | 1.12 | 1.88 | 1.20 | 1.04 | Discovery STE |
| 073_S_4393 | 1.17 | 0.99 | 1.15 | 1.77 | 1.18 | 1.02 | Discovery STE |
| 073_S_4552 | 1.15 | 0.98 | 1.11 | 1.68 | 1.18 | 1.04 | Discovery STE |
| 073_S_4739 | 1.06 | 1.01 | 1.12 | 1.61 | 1.05 | 0.94 | Discovery STE |
| 073_S_4762 | 1.13 | 0.99 | 1.13 | 1.64 | 1.14 | 1.00 | Discovery STE |
| 073_S_4795 | 1.14 | 1.01 | 1.19 | 1.77 | 1.13 | 0.96 | Discovery STE |
| 073_S_5023 | 1.22 | 1.01 | 1.15 | 1.80 | 1.20 | 1.06 | Discovery STE |
| 082_S_4090 | 1.55 | 1.15 | 1.37 | 2.41 | 1.35 | 1.13 | LSO HI-REZ (CPS) |
| 082_S_4208 | 1.22 | 1.06 | 1.20 | 1.86 | 1.16 | 1.02 | LSO HI-REZ (CPS) |
| 082_S_4224 | 1.40 | 1.12 | 1.35 | 2.13 | 1.25 | 1.04 | LSO HI-REZ (CPS) |
| 082_S_4428 | 1.38 | 1.07 | 1.25 | 1.94 | 1.30 | 1.11 | LSO HI-REZ (CPS) |
| 094_S_4234 | 1.18 | 0.99 | 1.15 | 1.75 | 1.20 | 1.03 | Siemens HR+ |
| 094_S_4503 | 1.18 | 0.96 | 1.09 | 1.83 | 1.23 | 1.08 | Siemens HR+ |
| 094_S_4649 | 1.04 | 0.90 | 1.03 | 1.53 | 1.15 | 1.00 | Siemens HR+ |
| 098_S_4506 | 1.22 | 1.04 | 1.20 | 1.88 | 1.17 | 1.01 | Discovery STE |
| 116_S_4043 | 1.03 | 0.95 | 1.09 | 1.56 | 1.09 | 0.95 | Discovery ST |
| 116_S_4092 | 1.52 | 1.07 | 1.21 | 1.94 | 1.43 | 1.26 | Discovery ST |
| 116_S_4453 | 1.15 | 0.99 | 1.15 | 1.72 | 1.16 | 1.00 | Discovery ST |
| 116_S_4483 | 1.08 | 1.01 | 1.12 | 1.54 | 1.07 | 0.97 | Discovery ST |
| 116_S_4855 | 1.49 | 1.03 | 1.20 | 1.85 | 1.45 | 1.24 | Discovery ST |
| 127_S_4604 | 1.31 | 1.03 | 1.18 | 2.00 | 1.27 | 1.10 | Siemens HR+ |
| 127_S_4645 | 1.42 | 1.01 | 1.16 | 1.98 | 1.41 | 1.22 | Siemens HR+ |
| 127_S_4843 | 1.56 | 1.08 | 1.24 | 2.26 | 1.44 | 1.25 | Siemens HR+ |
| 128_S_4586 | 1.47 | 1.18 | 1.33 | 2.11 | 1.25 | 1.10 | Siemens HRRT |
| Patient | **Cortex AVG** | **CGM AVG** | **WC AVG** | **WM AVG** | **SUVR_CWM_** | **SUVR_WC_** | **Scanner** |
| 128_S_4607 | 1.21 | 1.07 | 1.20 | 1.71 | 1.13 | 1.01 | Siemens HRRT |
| 128_S_4609 | 1.14 | 1.00 | 1.11 | 1.49 | 1.14 | 1.03 | Siemens HRRT |
| 129_S_4369 | 1.13 | 1.01 | 1.15 | 1.66 | 1.11 | 0.98 | Siemens HR+ |
| 129_S_4396 | 1.05 | 1.00 | 1.08 | 1.55 | 1.05 | 0.97 | Siemens HR+ |
| 129_S_4422 | 1.32 | 0.98 | 1.09 | 1.72 | 1.35 | 1.22 | Siemens HR+ |
| 130_S_4352 | 1.54 | 1.17 | 1.40 | 2.49 | 1.32 | 1.10 | Siemens Biograph 64 |
| 135_S_4446 | 1.20 | 1.00 | 1.14 | 1.82 | 1.19 | 1.05 | Siemens HR+ |
| 135_S_4566 | 1.38 | 1.02 | 1.18 | 2.03 | 1.36 | 1.18 | Siemens HR+ |
| 135_S_4598 | 1.27 | 0.96 | 1.13 | 1.99 | 1.33 | 1.13 | Siemens HR+ |
| 136_S_4269 | 1.28 | 1.08 | 1.22 | 1.91 | 1.18 | 1.05 | Discovery ST |
| 137_S_4466 | 1.09 | 1.01 | 1.11 | 1.61 | 1.08 | 0.98 | Discovery STE |
| 137_S_4482 | 1.88 | 1.11 | 1.28 | 2.28 | 1.69 | 1.46 | Discovery STE |
| 137_S_4587 | 1.62 | 1.03 | 1.22 | 2.15 | 1.58 | 1.33 | Discovery STE |
| 137_S_4632 | 1.11 | 0.95 | 1.11 | 1.70 | 1.17 | 1.00 | Discovery STE |
| 153_S_4125 | 1.18 | 1.08 | 1.22 | 1.82 | 1.10 | 0.97 | BioGraph TruePoint |
| 153_S_4151 | 1.80 | 1.10 | 1.31 | 2.38 | 1.63 | 1.37 | BioGraph TruePoint |
| 153_S_4372 | 1.33 | 1.10 | 1.26 | 2.04 | 1.21 | 1.05 | BioGraph TruePoint |
| 941_S_4100 | 1.41 | 0.96 | 1.06 | 1.68 | 1.46 | 1.33 | GEMINI TF TOF |
| 941_S_4255 | 1.39 | 1.06 | 1.23 | 2.05 | 1.32 | 1.13 | GEMINI TF TOF |
| 941_S_4365 | 1.40 | 0.99 | 1.16 | 2.03 | 1.41 | 1.21 | GEMINI TF TOF |
| 941_S_4376 | 1.04 | 1.01 | 1.09 | 1.37 | 1.03 | 0.95 | GEMINI TF TOF |
